# Lower limb biomechanics of fully trained exoskeleton users reveal complex mechanisms behind the reductions in energy cost with human-in-the-loop optimization

**DOI:** 10.3389/frobt.2024.1283080

**Published:** 2024-01-31

**Authors:** Katherine L. Poggensee, Steven H. Collins

**Affiliations:** ^1^ Department of Mechanical Engineering, Stanford University, Stanford, CA, United States; ^2^ Department of Rehabilitation Medicine, Erasmus MC, Rotterdam, Netherlands; ^3^ Faculty of Mechanical, Maritime and Materials Engineering (3mE), Technical University of Delft, Delft, Netherlands; ^4^ Department of Bioengineering, Stanford University, Stanford, CA, United States

**Keywords:** exoskeletons, biomechanics, gait, human-in-the-loop optimization, plantarflexion assistance

## Abstract

Exoskeletons that assist in ankle plantarflexion can improve energy economy in locomotion. Characterizing the joint-level mechanisms behind these reductions in energy cost can lead to a better understanding of how people interact with these devices, as well as to improved device design and training protocols. We examined the biomechanical responses to exoskeleton assistance in exoskeleton users trained with a lengthened protocol. Kinematics at unassisted joints were generally unchanged by assistance, which has been observed in other ankle exoskeleton studies. Peak plantarflexion angle increased with plantarflexion assistance, which led to increased total and biological mechanical power despite decreases in biological joint torque and whole-body net metabolic energy cost. Ankle plantarflexor activity also decreased with assistance. Muscles that act about unassisted joints also increased activity for large levels of assistance, and this response should be investigated over long-term use to prevent overuse injuries.

## 1 Introduction

Exoskeletons have emerged as a tool to improve locomotion. Walking and running can be made easier by wearing assistive devices at the ankles (Malcolm et al., 2013), knees (MacLean and Ferris, 2019), hips (Young et al., 2017; Kim et al., 2019), and any combination of these joints (Lee et al., 2018; Franks et al., 2021). Exoskeletons can also be used in rehabilitation to offset the effects of movement disorders (Awad et al., 2020; Orekhov et al., 2020).

People alter their gait in stereotyped ways to take advantage of the exoskeleton assistance. Plantarflexion assistance typically results in increased peak plantarflexion angle (Sawicki and Ferris, 2008; Galle et al., 2013; Mooney and Herr, 2016; Koller et al., 2018) with a reduced dorsiflexion during stance (Sawicki and Ferris, 2008; Mooney and Herr, 2016; Koller et al., 2018; Grimmer et al., 2019) and no change in kinematics at the unassisted joints (Gordon and Ferris, 2007; Sawicki and Ferris, 2008; Galle et al., 2013; Jackson and Collins, 2015; Mooney and Herr, 2016; Koller et al., 2018; Grimmer et al., 2019). Exoskeleton use leads to reductions in biological power at the assisted joints (Sawicki and Ferris, 2008; Collins et al., 2015; Jackson and Collins, 2015; Mooney and Herr, 2016; Koller et al., 2018; Grimmer et al., 2019). Muscle activity at the assisted joints decreases (Gordon and Ferris, 2007; Sawicki and Ferris, 2008; Galle et al., 2013; Collins et al., 2015; Jackson and Collins, 2015; Koller et al., 2018; Moltedo et al., 2020; Wang et al., 2020), accompanied by co-contraction of the antagonist muscles (Sawicki and Ferris, 2009; Galle et al., 2013; Collins et al., 2015; Jackson and Collins, 2015) and some reduction in muscle activity at other joints (Gordon and Ferris, 2007; Galle et al., 2013; Koller et al., 2018).

Biomechanics typically also change as people adapt to new environments, such as assisted walking (Gordon and Ferris, 2007; Sawicki and Ferris, 2008). Coordination patterns have been shown to stabilize more quickly than metabolic cost (Huang et al., 2012), and muscle activity in the assisted joints is reduced shortly after the introduction of assistance (Abram et al., 2022), but the exact adaptation timelines are unknown. We previously conducted a study in which energy cost was significantly reduced with bilateral ankle exoskeleton assistance (Poggensee and Collins, 2021), which was achieved after adaptation over 109 min of walking. Participants walked with either a static, unchanging assistance profile or in an optimization protocol, and, for the same condition, reduced the energy cost of walking by 28% and 31% compared to an unpowered condition. With an optimized profile, participants further reduced energy expenditure, resulting in a reduction of 39% of the energy cost of the unpowered condition. Given the slow nature of locomotor adaptation and large changes in energy economy, the biomechanics that have previously been reported may represent a locally optimal coordination pattern rather than a globally optimal gait. A similar phenomenon was observed in split-belt treadmill walking (Sánchez et al., 2021).

The purpose of this study is twofold. First to explain the mechanisms for the reduction in energy cost. Metabolic cost, muscle activity, and joint mechanics are not always measured in parallel (Gordon and Ferris, 2007; Zhang et al., 2017; Wang et al., 2020), so what drives the reduction in energy cost in assisted walking is still an open question. Sources include a reduction in active muscle volume, which correlates with improved locomotion economy (Beck et al., 2019), or a reduction in biological mechanical power (Mooney and Herr, 2016). Furthermore, this parameterization has resulted in larger energetic benefits than previous iterations, but the biomechanical responses have only been partially examined (Zhang et al., 2017; Abram et al., 2022). The second purpose for this study is to characterize the biomechanics of adapted exoskeleton walking for a simple time-based torque trajectory. We will use a subset of the data from Poggensee and Collins (2021), limiting analysis to the experimental groups that fully adapted to exoskeleton assistance.

## 2 Materials and methods

### 2.1 Description of the experimental protocol behind the dataset

The prior experiment which generated the data used in this study is explained in full detail in the paper by Poggensee and Collins (2021), but the relevant details are described here. Of the fifteen participants tested in the first experiment, this study is limited to the two groups that demonstrated full adaptation to the device, i.e., the groups trained with moderate to no variation. Similarly, this study was limited to the validation tests on the final day to standardize analysis across participants and ensure full adaptation. Ten novice participants learned to walk with ankle exoskeletons. One group (*static training*; 2 F/3 M, 23.6 ± 3.5 years, 1.7 ± 0.1 m, 67.5 ± 14.4 kg) experienced a static profile throughout the training period, and another experienced human-in-the-loop optimization (*continued optimization*; 2 F/3 M, 23.4 ± 1.1 years, 1.7 ± 0.1 m, 68.4 ± 8.6 kg). Participants all provided informed consent, and the study was approved by the Stanford University Institutional Review Board.

**FIGURE 1:**
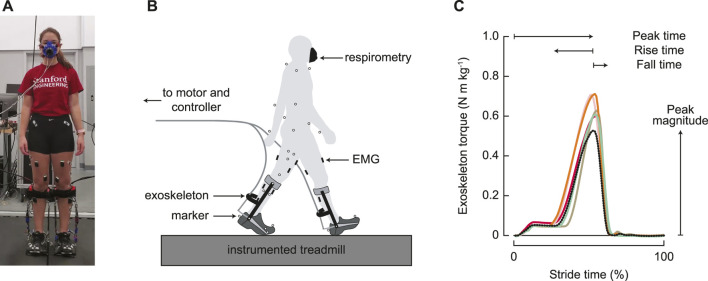
Experimental setup. **(A)** Participants wore bilateral ankle exoskeletons and walked on a split-belt, instrumented treadmill. **(B)** Metabolic energy cost, motion capture, ground reaction forces, and electromyography were measured throughout the experiment. **(C)** Exoskeleton control was parameterized by four parameters: magnitude of the peak torque; timing of the peak torque; rise time, or the duration of increasing torque before the peak; and fall time, or the duration of decreasing torque from the peak. All timing parameters were determined as a percentage of the stride time. The black dotted line represents the generic assistance condition, and the solid colored lines represent optimized profiles for the five individual participants in the group that experienced the optimization protocol.

The exoskeleton emulator system (shown in Figure 1A) was created in-house and has been extensively validated (Witte and Collins, 2020). The exoskeletons (0.88 kg each) consisted of a carbon fiber frame, attached to a commercially available running shoe, in men’s size 7, 9, 11, or 13. The exoskeletons were controlled to follow a time-based torque trajectory determined by four parameters (Figure 1C): the *peak magnitude* normalized to body mass; *peak time*, or when the peak occurred; *rise time*, or when the torque began to rise from zero; and *fall time*, or the time to decrease the torque to zero. All timing parameters were taken as a percentage of stride. This parameterization has been proven to be effective for reducing the energy cost of walking, at torque levels well within the operating range of the device (Zhang et al., 2017).

Metabolic power, motion capture, ground reaction force, and electromyography data were collected every day of the experiment (Figure 1B). Metabolic power was measured via an indirect calorimetry device (Cosmed Quark CPET, Rome, Italy) and computed using a standard equation (Brockway, 1987). Motion capture data were recorded at 100 Hz using a custom 55 marker set, which contained the markers from the Vicon Plug-in Gait model (Vicon, Oxford Metrics, Oxford, United Kingdom). The additional markers included markers placed on the upper body at the suprasternal notch, on the C7 vertebra, and on the shoulder, elbow, and wrist joints; individual markers on the greater trochanter and the fifth metatarsal; thigh and shank triad plates; and markers placed on the exoskeleton at the calf cuff attachment, the device ankle joint, and the device toe joint. These markers were used to improve tracking, but the Plug-in Gait model was used for inverse dynamics calculations. Ground reaction forces and moments were measured at 1,000 Hz using an instrumented split-belt treadmill (Bertec, Columbus, OH, United States). Lower limb muscle activity was measured for eight muscles on each leg using surface electromyography. Wireless electrodes were placed on the lateral aspect of the soleus, on the medial and lateral aspects of the gastrocnemius, and on the rectus femoris (Trigno Wireless system, Delsys, Boston, MA, United States). Mini wireless electrodes were placed on the tibialis anterior, vastus medialis, biceps femoris, and semitendinosus, with the electrode heads on the muscles of interest and the body on a neighboring muscle (Trigno Mini, Delsys, Boston, MA, United States). Voltages were recorded with motion capture at 1,000 Hz, and then were downsampled to 100 Hz.

The analysis presented here is limited to the performance in the validation tests on the final day of training; for more explanation on the training protocols, we refer the reader to the original paper (Poggensee and Collins, 2021). Participants experienced the following conditions:

• Normal walking (NW): a baseline condition, in which participants wear commercially available running shoes;
• Zero torque (ZT): an unpowered baseline condition, in which participants wear the exoskeletons in a transparent tracking mode;
• Generic assistance (GA): an assisted condition with a standardized assistance profile, with a peak magnitude of 0.54 N m kg^−1^, peak time at 52.9% of stride, rise time of 26.2%, and a fall time of 9.8%; and
• Optimized profile (OP): an assistance profile that was optimized for each participant, only in the *continued optimization* group. This profile was the result of twenty generations of optimization, with an objective to reduce the energy cost of walking.

Each condition was experienced for two 6-min trials in a block randomized ABC(DD)CBA order. For all conditions, participants walked on an in-ground treadmill (Bertec, Columbus, OH, USA) at a constant speed of 1.25 ms^−1^. With the exception of device work, none of the biomechanics results were analyzed in the initial study.

### 2.2 Data analysis

#### 2.2.1 Joint mechanics and stride time

Marker and analog trajectories were filtered using a fourth-order, low-pass Butterworth filter using 6 Hz and 300 Hz cut-off frequencies, respectively. Inverse kinematics and dynamics were computed using the Dynamic Plug-in Gait Pipeline (Vicon, Oxford Metrics, Oxford, United Kingdom), which produced joint kinematics, moments, and powers. Individual trials were excluded if the Plug-in Gait Pipeline failed to produce accurate trajectories. Net work was computed as the integral of joint power (*P*_joint_) over the entire stride (from heel strike *h* at time *t*_*h*_ to the following heel strike *h* + 1 at time *t*_*h*+1_),

$$\text{Joint net work rate}=\int_{h}^{h+1}P_{\text{joint}}/\left(t_{h+1}-t_{h}\right).$$ (1)

Heel strikes were determined by heel switches for exoskeleton measurements and vertical ground reaction force for joint mechanics. Stride time was computed as the time between ipsilateral heel strikes. Data were manually validated to remove erroneously identified heel strikes.

Exoskeleton kinematics and torque were measured by sensors onboard the device. An optical rotary encoder (Renishaw, Gloucestershire, United Kingdom) measured angular velocity, and strain gauges were used to measure torque from the device. Device power (*P*_exo_) was computed as

$$P_{\mathrm{exo}}=\tau_{a}\cdot {\dot{\theta }}_{a},$$ (2)

where *τ*_*a*_ is the measured ankle torque and ${\dot{\theta }}_{a}$ the measured ankle angular velocity. All exoskeleton measurements were recorded at 500 Hz using the real-time computer that controlled the device (Speedgoat, Liebefeld, Switzerland). For most participants, a time-sync signal was transmitted from the motion capture software to the real-time computer; for the datasets that did not include this measurement, the data were synced by heel strike. Biological ankle moments and powers were calculated by subtracting the measured exoskeleton mechanics from the total mechanics produced by the inverse dynamics pipeline.

#### 2.2.2 Muscle activity

The linear envelope of the electromyography signal was computed by first high-pass filtering the signals with a cut-off frequency of 30 Hz, rectifying, and then low-pass filtering with a cut-off frequency of 6 Hz (Jackson and Collins, 2015). All signals were normalized by the peak value of the average trajectory observed during normal walking and averaged across legs.

#### 2.2.3 Statistical analysis

Biomechanical trajectories were averaged over the final 3 min in each trial across participants. Trajectories were normalized to percent stride. Bounds for the time-based trajectories were determined by bootstrapping, with 1000 samples, in a similar manner to what is described in Poggensee and Collins (2021).

We computed points of interest for each individual participant, rather than the aggregated trajectories, to describe the effect of the different conditions. The kinematic points of interest included peak hip extension and hip angle at heel strike; peak knee flexion in stance and swing and extension in stance; peak ankle dorsiflexion and plantarflexion angles. Peak extension hip and knee moments as well as net hip and knee work were computed, as were peak total, biological, and exoskeleton plantarflexor moments. Peak positive and negative ankle power and net work rate were calculated for the total, biological, and device components. Per participant, per variable, there were up to four repeated measures, as a result of the two repeated trials and the bilateral exoskeletons. Therefore, up to forty points were obtained for each condition. Some variables (namely, hip and knee moments and powers, and left hip/knee angles for one participant) had fewer than forty measures in a condition due to incorrect inverse dynamics results. We tested for changes in these points of interest across the three device conditions (zero torque mode, generic assistance, and optimized assistance) by performing a one-way repeated measures ANOVA (with subject as a random effect), followed by *post hoc* multiple comparison analysis (Tukey HSD) to determine pairwise differences for models deemed significant for *α* = 0.05. Partial eta squared (*η* ^2^) was computed to determine the effect size of the ANOVA.

## 3 Results

### 3.1 Kinematics

**FIGURE 2:**
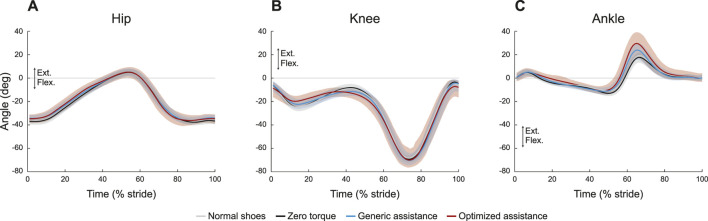
Joint kinematics profiles for the **(A)** hip, **(B)** knee, and **(C)** ankle. All profiles are normalized to stride period, defined by heel strike to ipsilateral heel strike, and averaged across participants. The gray lines represent walking with normal shoes; the black lines represent the unpowered, zero torque condition; the blue lines represent walking with generic assistance; and the red lines represent walking with an individual’s optimized assistance profile. The shaded regions around the three device conditions represent the upper and lower bounds determined by bootstrapping. Only half of the participants analyzed in this study had an optimized profile, so those trajectories are averaged over five participants instead of ten. For all measures, extension is positive.

Wearing the device in an unpowered zero torque mode did not change kinematics compared to normal walking, for any joint (Figure 2), although assistance slightly altered hip and knee kinematics (Table 1). Peak hip extension (*F* = 1.13, *p* = 0.27) did not exhibit significant changes across conditions, and, compared to zero torque, the hip angle at heel strike increased only with generic assistance, by 2.8° (*F* = 6.35, *p* = 0.003; pairwise comparison, *p* = 0.001). Knee kinematics did not change during swing (*F* = 1.07, *p* = 0.35). Knee flexion during early stance increased with assistance (*F* = 4.64, *p* = 0.01), increasing by 2.3° with generic assistance and by 2.5° with optimized assistance, compared to the unpowered condition. The knee was more flexed in assisted conditions during loading in the middle of stance (*F* = 5.95, *p* = 0.004), decreasing by 3.0° and 3.6° with generic and optimized assistance, respectively.

**TABLE 1:** Statistical tests on points of interest. The *F*-statistic and the corresponding *p*-value are presented for all ANOVA models with the effect size (*η* ^2^), with the *p* values from the corresponding *post hoc* pairwise comparison tests for significant ANOVA results. All variables in this table are the peak value unless specified; values that occur not at the peaks are shown in italicized font. Bolded values are statistically significant. Tot. = total, Bio. = biological.

		ANOVA			Post-hoc pairwise comparisons		
		*F*	*p*	*η* ^2^	GA vs. ZT	OP vs. ZT	OP vs. GA
Joint Kinematics	Hip extension	1.13	0.27	0.03			
	*Hip angle at heel strike*	6.35	**0.0027**	0.13	**0.001**	0.29	0.29
	Knee flexion (stance)	4.64	**0.012**	0.10	**0.029**	**0.03**	0.56
	Knee extension (stance)	5.95	**0.0039**	0.13	**0.0086**	**0.014**	0.58
	Knee flexion (swing)	1.07	0.35	0.03			
	Ankle dorsiflexion	4.55	**0.013**	0.09	**0.0091**	0.20	0.55
	Ankle plantarflexion	63.09	**<0.0001**	0.59	**6.2e-13**	**<2.2e-16**	**3.0e-7**
Ankle Kinetics	Tot. ankle moment	0.38	0.68	0.01			
	Bio. ankle moment	73.59	**<0.0001**	0.63	**<2.2e-16**	**<2.2e-16**	0.50
	Exoskeleton torque	121.99	**<0.0001**	0.80	N/A	N/A	**<2.2e-16**
	Tot. ankle power	28.24	**<0.0001**	0.40	**1.9e-6**	**4.2e-12**	**0.0011**
	Bio. ankle power	1.68	0.19	0.04			
	Exoskeleton power	49.19	**<0.0001**	0.61	N/A	N/A	**2.3e-12**
	Negative tot. ankle moment	23.37	**<0.0001**	0.35	**2.6e-11**	**0.0051**	**0.015**
	Negative bio. ankle moment	53.14	**<0.0001**	0.56	**<2.2e-16**	**3.2e-8**	**0.020**
Joint Work	Hip net work rate	5.39	**0.0065**	0.12	**0.0036**	0.18	0.54
	Knee net work rate	4.70	**0.012**	0.11	**0.0066**	0.39	0.39
	Tot. ankle net work rate	89.95	**<0.0001**	0.51	**<2.2e-16**	**<2.2e-16**	**7.3e-6**
	Bio. ankle net work rate	15.92	**<0.0001**	0.27	**3.2e-5**	**1.8e-6**	0.12
Muscle Activity	Soleus	160.60	**<0.0001**	0.79	**<2.2e-16**	**<2.2e-16**	**0.0090**
	Medial gastrocnemius	50.59	**<0.0001**	0.54	**5.4e-11**	**<2.2e-16**	**6.2e-6**
	Lateral gastrocnemius	92.20	**<0.0001**	0.68	**<2.2e-16**	**<2.2e-16**	**0.96**
	Tibialis anterior (stance)	1.52	0.22	0.03			
	Tibialis anterior (swing)	3.15	**0.048**	0.07	0.23	**0.039**	0.23
	Rectus femoris	12.47	**<0.0001**	0.22	**0.019**	**0.00014**	**2.13e-6**
	Vastus medialis	9.29	**2.0e-4**	0.18	**5.0e-5**	0.15	0.16
	Semitendinosus	11.23	**<0.0001**	0.21	**0.017**	**6.9e-6**	**4.1e-4**
	Biceps femoris	29.05	**<0.0001**	0.40	**0.034**	**4.6e-13**	**4.8e-11**
	*Biceps femoris at heel strike*	1.17	0.31	0.03			

Ankle kinematics exhibited the largest change in the kinematics of any joint (Figure 2C; Table 1). Peak plantarflexion changed with assistance (*F* = 63.1, *p* < 0.0001; GA-ZT, *p* = 6.2e-13; OP-ZT, *p* < 2e-16; OP-GA, *p* = 3e-7), from 18° with zero torque, to 25° with generic assistance, and to 32° with optimized assistance. Peak dorsiflexion decreased slightly (*F* = 4.6, *p* = 0.01) from 13° with zero torque to 11° with both types of assistance. Only the generic assistance condition was significantly lower than the zero torque condition (*p* = 0.009; OP-ZT, *p* = 0.20).

**FIGURE 3:**
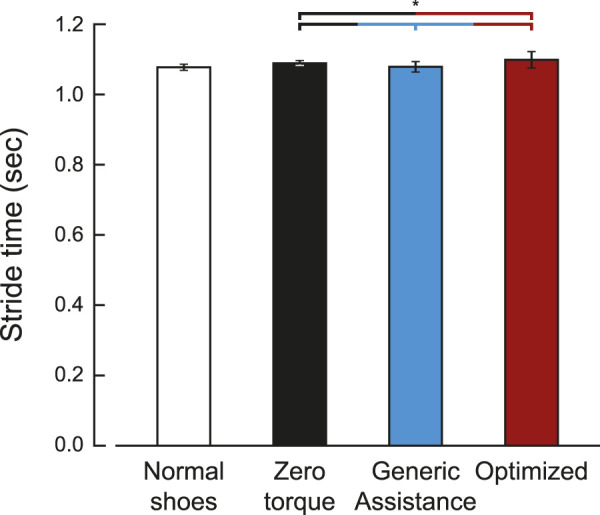
Stride time for each condition. Stride time was defined by the period between ipsilateral heel strikes. The optimized stride time is averaged over five participants, as five of the ten participants did not have an optimized assistance profile. Bars are the average stride time, and the error bars are one standard deviation.

Differences in stride times across condition were small but statistically significant (Figure 3; *F* = 280, *p* < 0.001). Stride time increased in the zero torque condition (1.08 ± 0.06 s) compared to the normal walking condition (1.07 ± 0.06 sec, *p* < 0.001). Generic assistance caused participants to walk with a decreased stride time compared to the zero torque condition (1.07 ± 0.06 sec, *p* < 0.001), and optimized assistance had the opposite effect (1.09 ± 0.07 sec, *p* = 0.004).

### 3.2 Joint moments and powers

**FIGURE 4:**
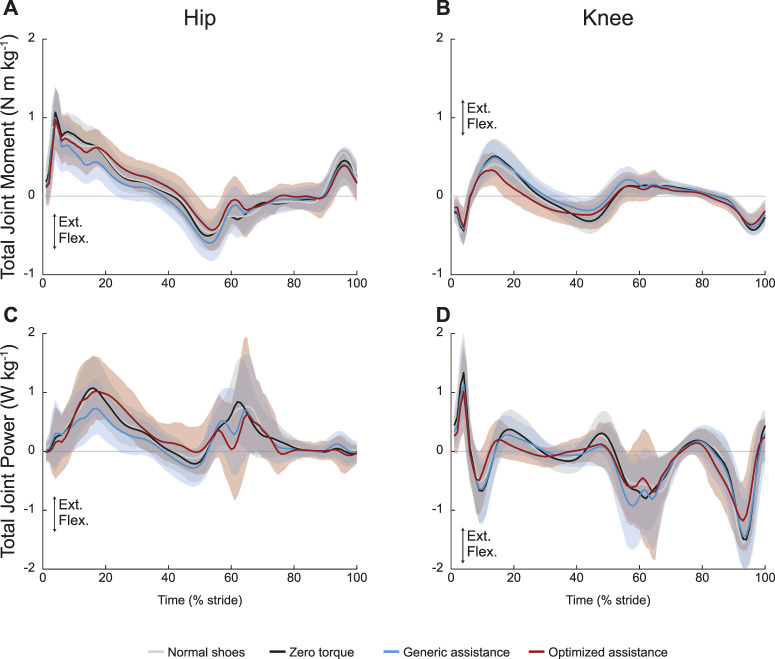
Unassisted joint mechanics: **(A)** hip and **(B)** knee total joint moments and **(C)** hip and **(D)** knee total joint power. All profiles are normalized to stride period, defined by heel strike to ipsilateral heel strike, and averaged across participants. The gray lines represent walking with normal shoes; the black lines represent the unpowered, zero torque condition; the blue lines represent walking with generic assistance; and the red lines represent walking with an individual’s optimized assistance profile. The shaded regions around the three device conditions represent the upper and lower bounds determined by bootstrapping. Only half of the participants analyzed in this study had an optimized profile, so those trajectories are averaged over five participants instead of ten. For all measures, extension is positive.

Peak joint moments showed no change across conditions at the hip (Figure 4A; *F* = 1.11, *p* = 0.33) and at the knee (Figure 4B; *F* = 2.2, *p* = 0.12). At an individual level, there appear to be changes in knee kinetics, but the varied personalized responses obscured group-level changes. Total joint power for the unassisted joints are shown in Figures 4C and D, respectively.

The ANOVA results were statistically significant for net work rate at both the hip and knee, but only the generic assistance condition resulted in any significant change. At the hip (*F* = 5.4, *p* = 0.007), net work rate decreased by 28% from the zero torque condition to the generic assistance condition (*p* = 0.004) and did not change with optimized assistance (*p* = 0.18). At the knee (*F* = 4.7, *p* = 0.012), net work rate decreased from −0.13 J kg^−1^ s^−1^ in the zero torque condition to −0.19 J kg^−1^ s^−1^ in the generic assistance condition (*p* = 0.007); optimized assistance did not significantly change net knee work rate (*p* = 0.39).

**FIGURE 5:**
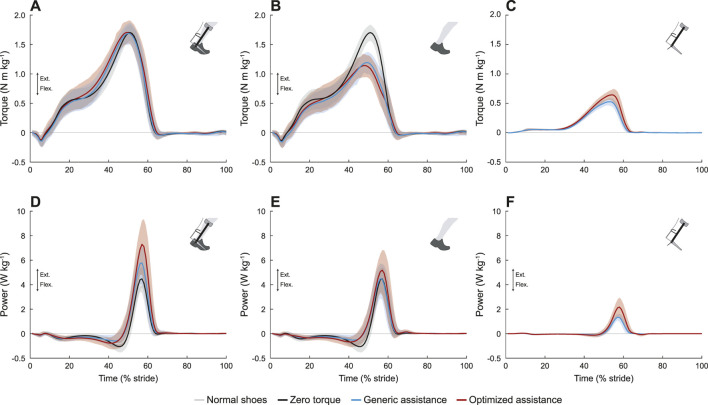
Ankle joint mechanics. **(A)** Total torque. The total ankle moment was unchanged with assistance. **(B)** Biological torque, including the zero torque condition. Peak biological torque decreased with assistance. **(C)** Exoskeleton torque. The peak exoskeleton torque was higher in the optimized condition. **(D)** Total ankle joint power. The peak positive power at the ankle increased with assistance, and with an even greater increase for the optimized condition. The peak negative power was reduced with assistance. **(E)** Biological ankle power, including zero torque. Peak positive biological ankle power was unchanged with assistance, but peak negative biological ankle power was reduced with assistance. **(F)** Exoskeleton power. Peak exoskeleton power was greater for optimized assistance compared to the generic profile. All profiles are normalized to stride period, defined by heel strike to ipsilateral heel strike, and averaged across participants. The gray lines represent walking with normal shoes; the black lines represent the unpowered, zero torque condition; the blue lines represent walking with generic assistance; and the red lines represent walking with an individual’s optimized assistance profile. The shaded regions around the three device conditions represent the upper and lower bounds determined by bootstrapping. Only half of the participants analyzed in this study had an optimized profile, so those trajectories are averaged over five participants instead of ten.

Device and biological ankle moments changed with assistance (Figures 5B, C; *F* = 122, *p* < 0.0001, and *F* = 74, *p* < 0.0001, respectively), but the total ankle moment was constant (Figure 5A; *F* = 0.38, *p* = 0.69). Peak device torque increased from generic assistance, 0.53 N m kg^−1^, to an average of 0.65 N m kg^−1^ for optimized assistance (*p* < 2.2e-16). Biological torque therefore decreased with assistance: compared to a peak ankle moment of 1.69 N m kg^−1^ in the zero torque condition, generic assistance reduced the biological ankle moment by 28% to 1.21 N m kg^−1^ (*p* < 2.2e-16), and optimized assistance reduced it by 31% to 1.17 N m kg^−1^ (*p* < 2.2e-16). Biological torque was statistically similar between assisted conditions (*p* = 0.50).

Total and biological negative ankle power decreased with assistance. Total negative power changed with assistance (Figure 5D; *F* = 23, *p* < 0.0001), decreasing from −1.15 N m kg^−1^ in the zero torque condition to −0.75 N m kg^−1^ with generic assistance (34% reduction, *p* = 2.6e-11), and with optimized assistance to −0.95 N m kg^−1^ (18% reduction, *p* = 0.005). Total negative power with generic assistance was significantly lower than for optimized assistance (*p* = 0.015). Decreases in negative biological power were even more pronounced (Figure 5E; *F* = 53, *p* < 0.0001). Negative biological ankle power decreased with generic assistance to −0.64 N m kg^−1^ (44% reduction, *p* < 2.2e-16) and to −0.81 N m kg^−1^ with optimized assistance (29% reduction, *p* = 3.2e-8), compared to the zero torque condition; generic assistance resulted in a greater decrease in negative biological ankle power than optimized assistance (*p* = 0.02). Negative power from the exoskeleton was negligible in all conditions (Figure 5F).

Peak positive total ankle power increased with assistance (Figure 5D; *F* = 28, *p* < 0.0001). Peak total positive ankle power was 4.62 W kg^−1^ in the zero torque condition, and increased to 5.95 W kg^−1^ with generic assistance (29% increase, *p* = 1.9e-6). Optimized assistance resulted in the highest peak total ankle power at 7.45 W kg^−1^, increasing by 61% compared to zero torque (*p* = 4.2e-12) and by 25% compared to generic assistance (*p* = 0.001).

Any condition-level differences in peak biological ankle power were not statistically significant (Figure 5E; *F* = 1.7, *p* = 0.19). While the peak biological ankle power during the generic assistance condition (4.7 W kg^−1^) was visually similar to the peak ankle power measured during the zero torque condition, the slight increase to 5.4 W kg^−1^ with optimized assistance was not significant due to intersubject variability.

Net ankle work rate also increased with assistance (Table 1; *F* = 89.9, *p* < 0.0001, total; *F* = 15.9, *p* < 0.0001, biological). Compared to the zero torque condition, total net ankle work rate increased 99.2% with generic assistance (*p* < 2.2e-16) and 178% with optimized assistance (*p* < 2.2e-16); this also represented a 40% increase from the generic assistance condition to the optimized assistance condition (*p* = 7.3e-6). Biological net ankle work also increased, compared to the zero torque condition, by 55% with generic assistance (*p* = 3.2e-5) and by 88% with optimized assistance (*p* = 1.8e-6), although there was no difference between the assistance conditions (*p* = 0.12).

### 3.3 Muscle activity

**FIGURE 6:**
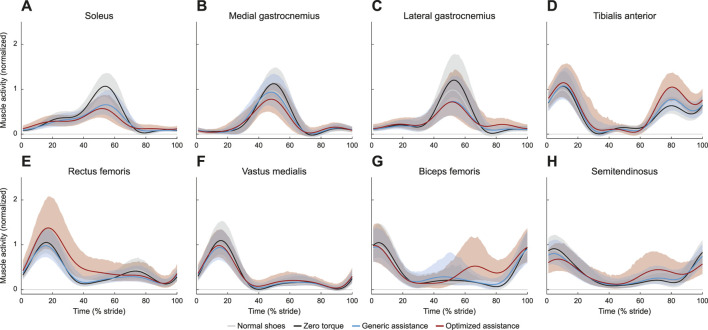
Muscle activity for **(A)** soleus, **(B)** medial aspect of the gastrocnemius, **(C)** lateral aspect of the gastrocnemius, **(D)** tibialis anterior, **(E)** rectus femoris, **(F)** vastus medialis, **(G)** biceps femoris, and **(H)** semitendinosus. All profiles are normalized to stride period, defined by heel strike to ipsilateral heel strike, and to the peak voltage of the average normal walking profile for each muscle. Presented trajectories are averaged across subjects and over the right and left legs, with bounds determined by bootstrapping. The gray lines represent walking with normal shoes; the black lines represent the unpowered, zero torque condition; the blue lines represent walking with generic assistance; and the red lines represent walking with an individual’s optimized assistance profile.

Ankle plantarflexion assistance resulted in decreased plantarflexor activity, across the soleus (Figure 6A; *F* = 161, *p* < 0.0001), the medial gastrocnemius (Figure 6B; *F* = 51, *p* < 0.0001), and the lateral gastrocnemius (Figure 6C; *F* = 92, *p* < 0.0001). Peak soleus activity decreased by 38% with generic assistance (*p* < 2.2e-16) and by 45% with optimized assistance (*p* < 2.2e-16), compared to zero torque. Medial and lateral gastrocnemius followed similar trends. Compared to zero torque, peak medial gastrocnemius activity decreased by 16% with generic assistance (*p* = 5.4e-11) and by 30% with optimized assistance (*p* < 2.2e-16), and peak lateral gastrocnemius activity decreased by 38% with generic assistance (*p* < 2.2e-16) and by 35% for optimized assistance (*p* < 2.2e-16). The soleus (*p* = 0.009) and medial gastrocnemius (*p* = 6.2e-6) were furthered reduced with optimized compared to generic assistance, although peak lateral gastrocnemius activity remained unchanged (*p* = 0.96).

Tibialis anterior activity was characterized by two peaks, one during stance and one during swing (Figure 6D). The peak activity during stance was unchanged with assistance (*F* = 1.5, *p* = 0.22). During swing (*F* = 3.2, *p* = 0.048), peak tibialis anterior activity increased by 72% with optimized assistance (*p* = 0.04), but was unchanged with generic assistance (*p* = 0.23).

Muscles acting about the knee were all affected by condition. Peak rectus femoris activity in stance increased with optimized assistance, by 36% compared with the zero torque condition (Figure 6E; *p* = 1.4e-4) and by 49% compared to the generic torque condition (*p* = 2.1e-6). Peak vastus medialis activity decreased with generic assistance by 13% compared with zero torque (Figure 6F; *p* = 5.0e-5). Hamstrings activity increased during swing with optimized assistance. Biceps femoris and semitendinosus activity during swing increased by 188% and 108%, respectively, with optimized assistance, compared to zero torque (Figures 6G, H; *p* = 4.6e-13, *p* = 6.9e-6, respectively). Both measured hamstrings muscles also showed an increase in activity with optimized assistance compared to the generic assistance condition: peak biceps femoris activity by 142% (*p* = 4.8e-11) and peak semitendinosus activity by 65% (*p* = 4.1e-4). The ANOVA results for these and all point statistics are shown in Table 1.

## 4 Discussion

We characterized the biomechanical response to exoskeleton assistance and identified potential changes in biomechanics which could account for the improved energy economy in fully adapted users. Many of the patterns reported here have also been observed in participants with less ankle exoskeleton experience (Gordon and Ferris, 2007; Sawicki and Ferris, 2008; Galle et al., 2013; Collins et al., 2015; Jackson and Collins, 2015; Mooney and Herr, 2016; van Dijk et al., 2016; Koller et al., 2018; Grimmer et al., 2019; Moltedo et al., 2020).

### 4.1 Muscle activity and joint power at the assisted joints may explain part of the reduction in energy cost

Plantarflexor activity decreased with plantarflexion assistance, on similar levels to what is observed for other exoskeletons (Gordon and Ferris, 2007; Sawicki and Ferris, 2008; Galle et al., 2013; Jackson and Collins, 2015; Koller et al., 2018), even for control architectures which rely on some level of plantarflexor activity (Gordon and Ferris, 2007; Koller et al., 2018). Plantarflexor activity decreases rapidly (Gordon and Ferris, 2007; Galle et al., 2013; Abram et al., 2022), so it is unsurprising that this reduction in plantarflexor activity is observed across exposure levels. While a reduction in muscle activity is one possible source for the reduction in energy cost, the discrepancy between reduced muscle activity and no to little change in metabolic cost observed in these studies suggests that this is not the sole driver. Without musculoskeletal simulations or ultrasound measurements to determine the internal dynamics (Jackson et al., 2017), it is difficult to conclude how large of a role the changes in plantarflexor muscle activity have on metabolic cost, but these results suggest that, with enough training, participants can simultaneously reduce plantarflexor activity while reducing energy cost.

Peak positive total ankle power and net work increased with assistance, but to a much larger degree than for users who may have had less exoskeleton experience (Mooney and Herr, 2016; Koller et al., 2018; Grimmer et al., 2019). The increase in total positive power was partially due to the increase in device power. Increasing device power and work in order to reduce energy cost has been proposed as a design target for lower-limb exoskeletons (Sawicki and Ferris, 2008; Mooney and Herr, 2016; Grimmer et al., 2019), and simply comparing optimized assistance profiles to generic profiles corroborates this idea (Witte et al., 2020; Poggensee and Collins, 2021). Peak torque magnitude was slow to increase as people co-adapted with the device, indicating that people should be fully trained in exoskeleton walking in order to fully realize the beneficial aspects of increased device work.

Peak positive biological ankle power and net biological ankle work increased. In other exoskeleton systems wherein users may have had less experience, total positive power increased with assistance, but also resulted in a decreased biological contribution (Jackson and Collins, 2015; Mooney and Herr, 2016; Koller et al., 2018; Grimmer et al., 2019). Increasing biological ankle work with assistance, while simultaneously reducing energy cost, suggests that the mechanical energy from the ankle is beneficial in terms of whole body energy usage. This increase in biological ankle work was also coupled with a reduction in plantarflexor activity, indicating that the plantarflexors may have operated more efficiently. Changes in muscle architecture were probably minimal given the relatively short duration of this experiment, so the increased efficiency in the muscle work would likely be the result of changes in the operating length and velocity of the muscle fibers themselves. Biological ankle work was unchanged across types of assistance, so increasing biological mechanical energy may have diminishing returns on improving energy economy.

Assistance and sufficient training may have resulted in more efficient gait, with a reduction in peak negative ankle power and in co-contraction. Increases in tibialis anterior activity have been reported, but those participants had significantly less exposure to ankle assistance (Galle et al., 2013; Jackson and Collins, 2015), reducing to levels seen in normal walking within an hour of training (Sawicki and Ferris, 2008). Co-contraction does aid motor adaptation by stiffening the joint and speeding the formation of an internal model for the novel paradigm, but decreases as the model accuracy improves (Osu et al., 2002; Heald et al., 2018), which could explain the increases in tibialis anterior activity seen in other exoskeleton studies. Reductions in peak negative ankle power or work has not been widely reported. We have observed very low levels of negative ankle work for this parameterization (Zhang et al., 2017; Witte et al., 2020), suggesting a negative correlation between negative ankle work and energetic benefits. We did not systematically vary levels of device work for this experienced population, so a more thorough characterization [such as Jackson and Collins (2015)] may better explain the relationship between external device work and metabolic energy.

With increasing levels of assistance, peak ankle plantarflexion angle, peak positive total ankle power, and peak net ankle work increased, while plantarflexor muscle activity decreased. Of these changes in ankle mechanics and motor control, peak plantarflexion angle was near the range of what was observed in previous exoskeleton studies (Gordon and Ferris, 2007; Sawicki and Ferris, 2008) and peak positive total ankle power was substantially higher than what has been observed (Gordon and Ferris, 2007; Mooney and Herr, 2016), which could indicate sources for the larger reductions in metabolic cost measured for these participants. Tibialis anterior muscle activity has been shown to decrease, i.e., co-contraction in the lower leg muscles decreases, as participants learn to use exoskeletons (Sawicki and Ferris, 2008), so these results indicate our participants are fully trained in the generic assistance condition and are therefore not increasing metabolic cost relative to unassisted walking. The increase in tibialis anterior activity during swing could serve as a mechanism to return the plantarflexed ankle to normal kinematics during early stance.

### 4.2 Unassisted joint biomechanics do not change in directions to reduce energy cost

The exoskeletons used in this study were limited to ankle plantarflexion assistance. This assistance profile was optimized and chosen to reduce the energy cost of walking and did not explicitly consider the impacts to the unassisted hip and knee joints.

The work done by the ankle may result in a beneficial transfer of energy to other joints, but not at the group level. While there was a change in net work rate across joints with assistance, these changes do not necessarily indicate the mechanism for the reduction in metabolic energy cost. In particular, during the optimized condition compared to the zero torque condition, although there was no change in summed hip and knee net work rate (−0.02 J kg^−1^ s^−1^), the biological net ankle work rate increased (0.14 J kg^−1^ s^−1^), which is counterintuitive to the large reduction in metabolic cost (−1.08 W kg^−1^).

The net work rate results for generic assistance are the most consistent across participants, which could be further proof that participants had fully learned this assistance profile even if they were still inexpert at the optimized profile. The summed hip and knee net work rate was almost entirely reduced to 0.02 J kg^−1^ s^−1^. These results only indicate the participants’ biomechanics on the final day of testing, but the previous study found a reduction in ankle work as participants gained experience with the device (Poggensee and Collins, 2021). Joint and muscle redundancy is beneficial for motor learning and could result in subject-specific mechanics while maintaining consistent end-effector (i.e., ankle) mechanics (Ranganathan and Newell, 2013; Singh et al., 2016). Finer analysis may reveal a variety of methods for transferring energy between joints and muscle groups (Siegel et al., 2004; Torres-Oviedo and Ting, 2010). Future study to understand the distribution of mechanical work across joints and how that distribution evolves as participants learn may result in another biomarker for tracking adaptation.

With generic assistance, there was a slight decrease in knee extensor activity during stance with no changes in knee flexor activity. Increases in vastus medialis activity have been reported in the contralateral knee for unilateral assistance torque (Jackson and Collins, 2015). The generic assistance torque in this study was within the range reported in Jackson and Collins (2015), so the reduction in activity was likely the result of training and not changes in assistance levels.

Knee muscle activity increased with optimized assistance, with the rectus femoris increasing during stance and the hamstrings increasing during swing. Hamstring activity decreased in other exoskeleton assistance studies (Galle et al., 2013; Jackson and Collins, 2015), even for assistance torques larger than our optimized profiles. Because the ankle is more plantarflexed at the end of stance and the tibialis anterior does not increase with assistance, the hamstrings may increase activity to allow for foot clearance during swing. Participants with the largest changes in rectus femoris activity had the highest optimized peak torque values, consistent with what has been observed for untrained unilateral exoskeleton users (Jackson and Collins, 2015). The energetic benefits from decreasing ankle muscle activity may outweigh the penalties imposed by increased knee muscle activity. By the nature of the optimization algorithm, participants have less exposure to the optimized parameters, so it is possible that prolonged training may reduce the rectus femoris activity observed here.

### 4.3 Exoskeleton assistance replaced the biological ankle moment to maintain an invariant total moment

Invariant ankle moment has been observed in participants trained for 1 hour, although those participants walked with much larger reductions in peak dorsiflexion angle compared to their baseline gait (Kao et al., 2010). Reductions in biological ankle moment have been observed for other devices, but often coupled with an increase in total ankle moment (Mooney and Herr, 2016; van Dijk et al., 2016; Grimmer et al., 2019).

Despite the consistent total ankle moment trajectory, peak ankle plantarflexion angle increased with assistance. The increase in peak plantarflexion angle at toe-off was consistent with other ankle exoskeleton studies (Gordon and Ferris, 2007; Sawicki and Ferris, 2008; Galle et al., 2013; Mooney and Herr, 2016; van Dijk et al., 2016; Koller et al., 2018; Grimmer et al., 2019), ranging from 5° to 20°. For both assistance profiles, large exoskeleton torques are applied at the end of stance, with the peak at 53% of stride for the generic assistance and at 54% for optimized assistance (Poggensee and Collins, 2021), which could explain the late-stance response to assistance. The change in plantarflexion angle with optimized assistance is at the higher end of the range seen for other participants. While there are scenarios where participants reduce their metabolic cost without substantially changing their ankle kinematics (Grimmer et al., 2019), large increases in plantarflexion angle, coupled with decreased plantarflexor activity, have been observed alongside higher reductions in metabolic cost (Koller et al., 2018). The increased plantarflexion angle, resulting from increased angular velocity at toe-off, coupled with decreased plantarflexor activity suggests that the reduction in metabolic cost can be explained by the calf muscles doing less work to move the ankle in an energetically-beneficial way.

Changes in peak dorsiflexion angle are observed across other exoskeleton studies, but with no consensus. While a reduction in dorsiflexion is most common (Gordon and Ferris, 2007; Sawicki and Ferris, 2008; Mooney and Herr, 2016; Koller et al., 2018; Grimmer et al., 2019), other devices have led to increased dorsiflexion (van Dijk et al., 2016; Moltedo et al., 2020). We observed decreased peak ankle dorsiflexion, but only in the generic assistance condition. The invariant ankle moment and minimal change in dorsiflexion angle suggest that the early stance mechanics in unassisted walking are energetically efficient, and that, with training, people can return to this gait.

### 4.4 Limitations and future work

While some of the deviations in exoskeleton-assisted gait from normal walking may explain the significant reductions in metabolic cost, the mechanisms behind the large improvement in energy economy cannot be fully defined by these data. For these trained participants, the ankle was plantarflexed more for similar reductions in plantarflexor activity compared to participants with less training. Training also resulted in less co-contraction and no change in total ankle moment. Assistance reduces active muscle volume by replacing some of the torque required at the ankle, which can lead to improvements in energy economy (Beck et al., 2019). While the increase in total power is consistent with other exoskeleton users, the simultaneous increase in biological ankle work, as well as the increase in knee muscle activity, would suggest an increase in metabolic cost. The work done by the ankle may result in a beneficial transfer of energy to other joints, but not at the group level. The increase in knee muscle activity is coupled with a decrease in ankle muscle activity, especially in biarticular muscles. The energetic benefits from decreasing ankle muscle activity may outweigh the penalties imposed by increased knee muscle activity.

We did not consider trunk or upper body biomechanics, nor how sagittal plane assistance affects the lower body biomechanics in other planes, for this study. We know that sagittal plane assistance can alter frontal plane dynamics (Kim and Collins, 2017). Analysis into these responses could lead to a better understanding of how people dynamically balance with these large assistive loads. There may also be reductions in muscles that were not covered in this study, such as the gluteus maximus, which may explain the improved energy economy. These results are also ambiguous when considering the different biomechanics observed for the well-trained generic assistance condition compared to the customized condition, under which users had less experience but exhibited greater metabolic cost reduction. Anecdotally, users reported a “lack of control” with the optimized condition, which may explain the varied responses, especially at the knee. Further research into how embodiment affects biomechanics and energy usage during gait could provide greater insight (Hybart and Ferris, 2023).

This study was limited by the narrow range of assistance strategies and small sample size. Full lower-limb exoskeletons would be better suited to tasks that require large energy reductions for locomotion. While the responses at the assisted joint may stay the same across devices, assistance at other joints may have implications in how loads are shared throughout the body. Multi-joint exoskeletons for able-bodied populations are rapidly improving (Lee et al., 2018; Bryan et al., 2020), and we would expect to see similar biomechanical characterizations as these devices become more ubiquitous. These data are freely available for further analysis (Poggensee and Collins, 2021). Increasing the pool of data available to study human responses to exoskeletons could lead to better understanding of how devices impact gait. Given the large costs necessary to train participants in exoskeleton gait, these biomechanical trajectories could augment strategies to rapidly identify beneficial devices and controllers, such as musculoskeletal simulations.

## 5 Conclusion

We characterized adapted biomechanical responses to different levels of plantarflexion assistance. The primary deviations from normal gait occurred at the assisted joint, with increased peak plantarflexion angle at toe-off, decreased peak biological ankle moment and plantarflexor activity, and increased biological ankle power, corresponding to decreased whole-body energy cost. Hip and knee kinematics were relatively unchanged, corroborating results seen in the literature, but were coupled with increases in muscle activity in the unassisted joints. While there was a decrease in joint work at the unassisted joints during generic assistance, the reductions in metabolic cost indicate more complex dynamics that cannot be fully explained by this analysis. These biomechanics profiles could be used as targets for training protocols, musculoskeletal simulations, or in device design. Future study on this subject is encouraged, especially involving other sensors to measure the internal musculoskeletal dynamics and varying device control as well as participant demographic parameters.

## Data Availability

The original contributions presented in the study are included in the article and in the dataset presented in Poggensee and Collins (2021). Further inquiries can be directed to the corresponding author.
